# Supplementary figures and images for: The associations between functional dyspepsia and potential risk factors: A comprehensive Mendelian randomization study
Source: PLoS One. 2024 May 8;19(5):e0302809. doi: 10.1371/journal.pone.0302809 (PMC11078438; doi:10.1371/journal.pone.0302809)

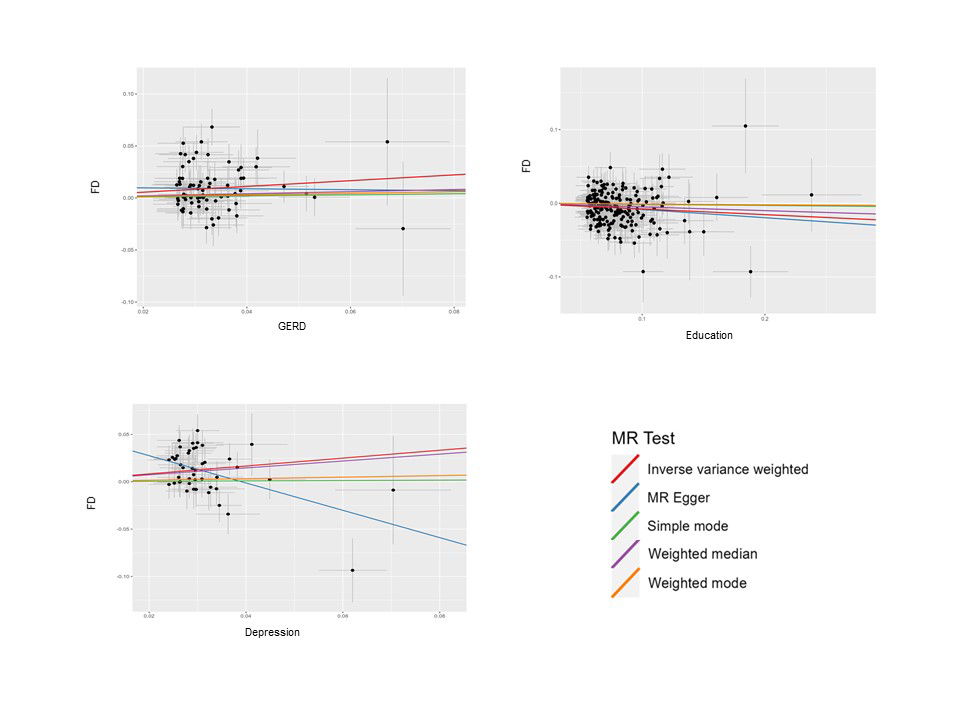

Supplement: S1 Fig — (TIF) [file pone.0302809.s001.tif]

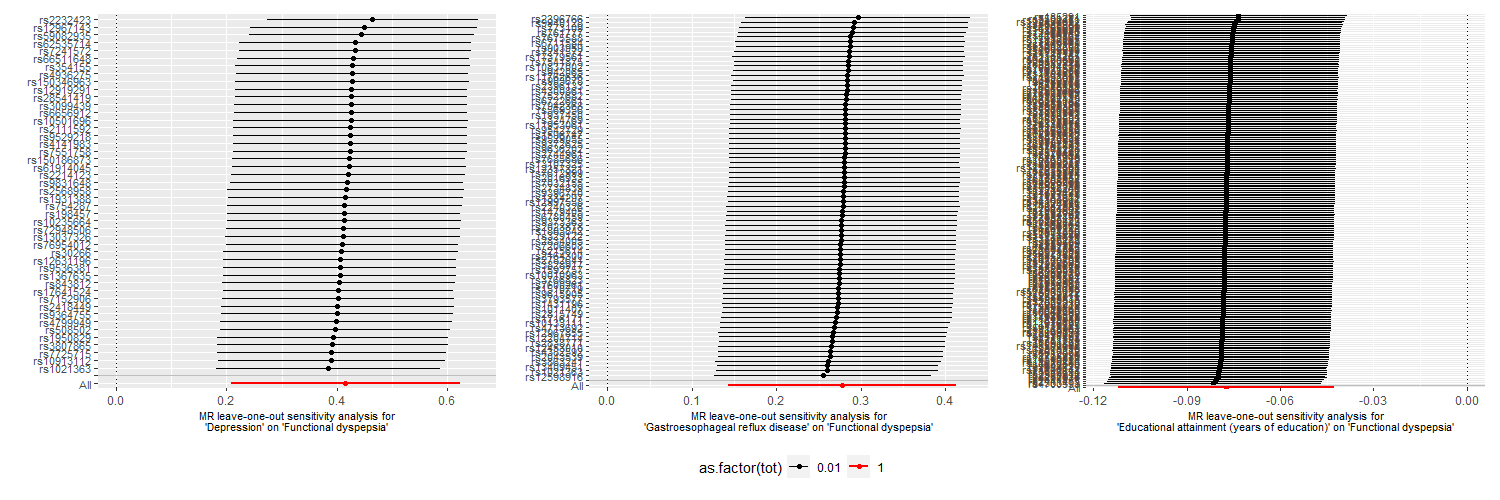

Supplement: S2 Fig — (TIF) [file pone.0302809.s002.tif]

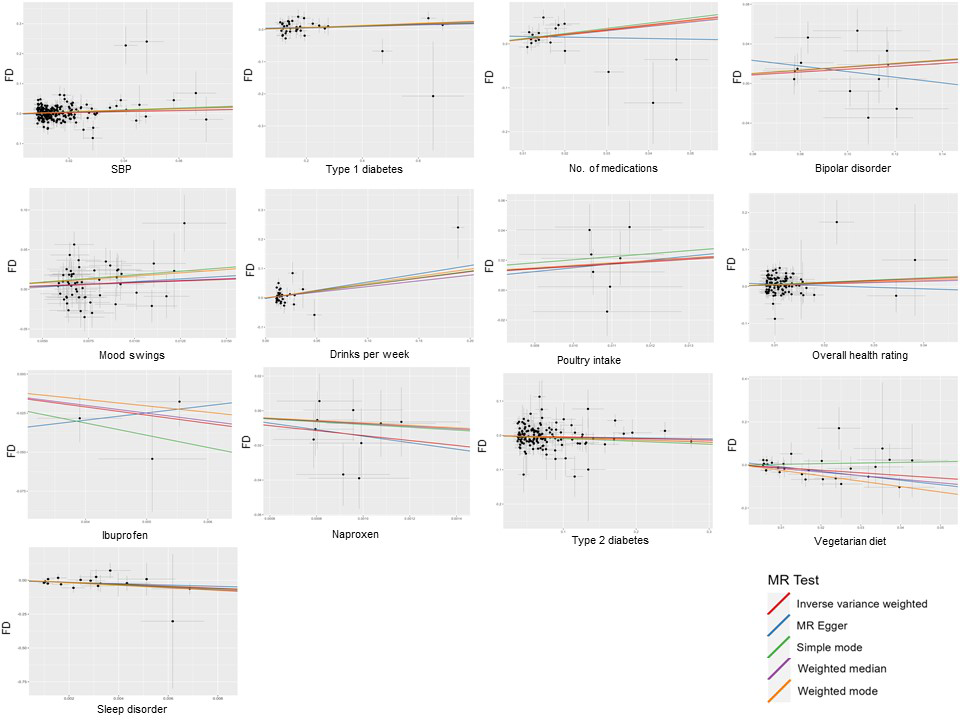

Supplement: S3 Fig — (TIF) [file pone.0302809.s003.tif]

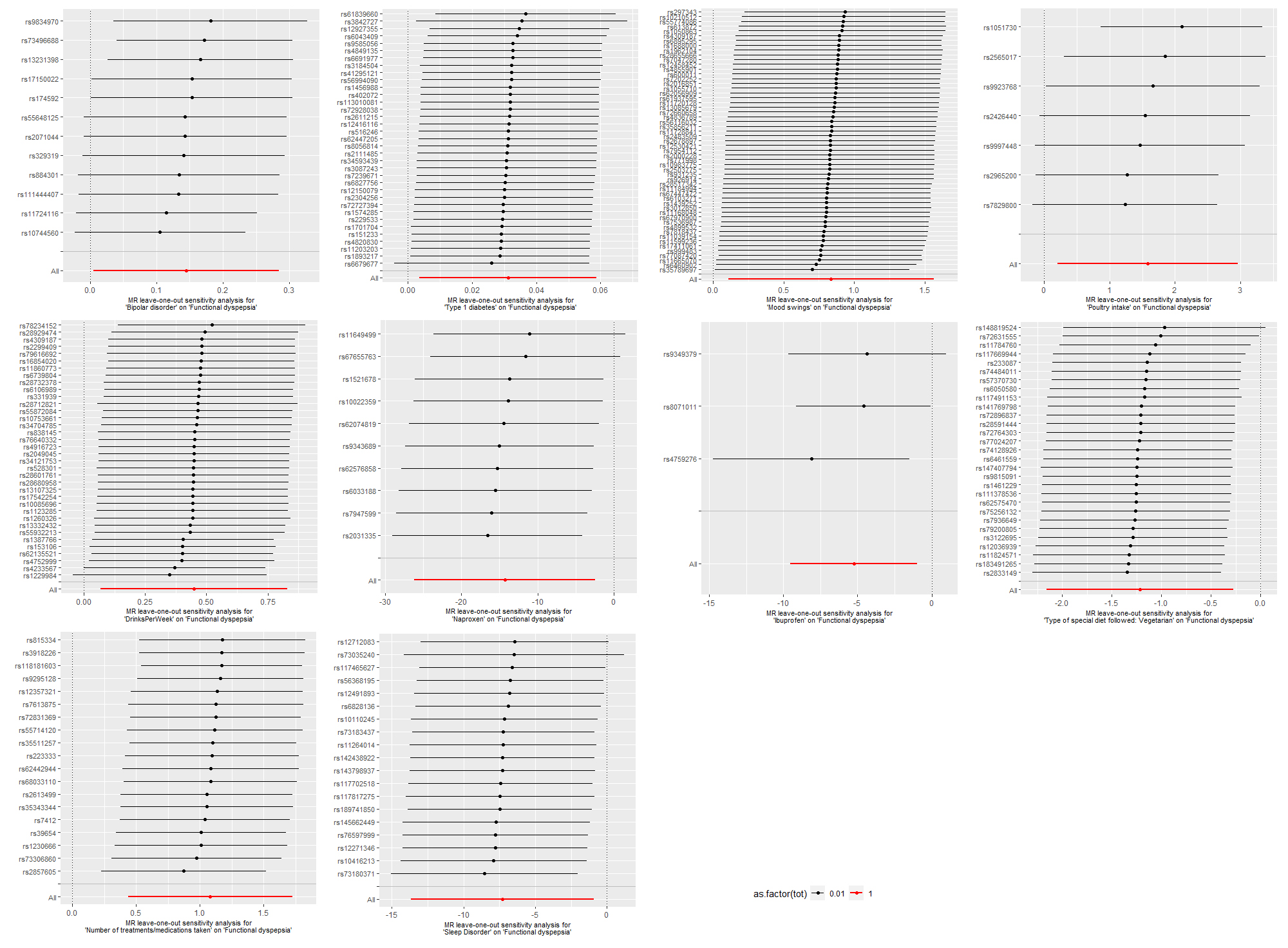

Supplement: S4 Fig — S4.1 Fig. Leave-one-out sensitivity test for suggestive factor (systolic blood pressure). S4.2 Fig. Leave-one-out sensitivity test for suggestive factor (type 2 diabetes). S4.3 Fig. Leave-one-out sensitivity test for suggestive factor (overall health rating). S4.4 Fig. Leave-one-out sensitivity test for suggestive factors (type 1 diabetes, bipolar disorder, mood swings, poultry intake, etc). (ZIP) [file pone.0302809.s004.zip › S4.4_Fig.TIF]

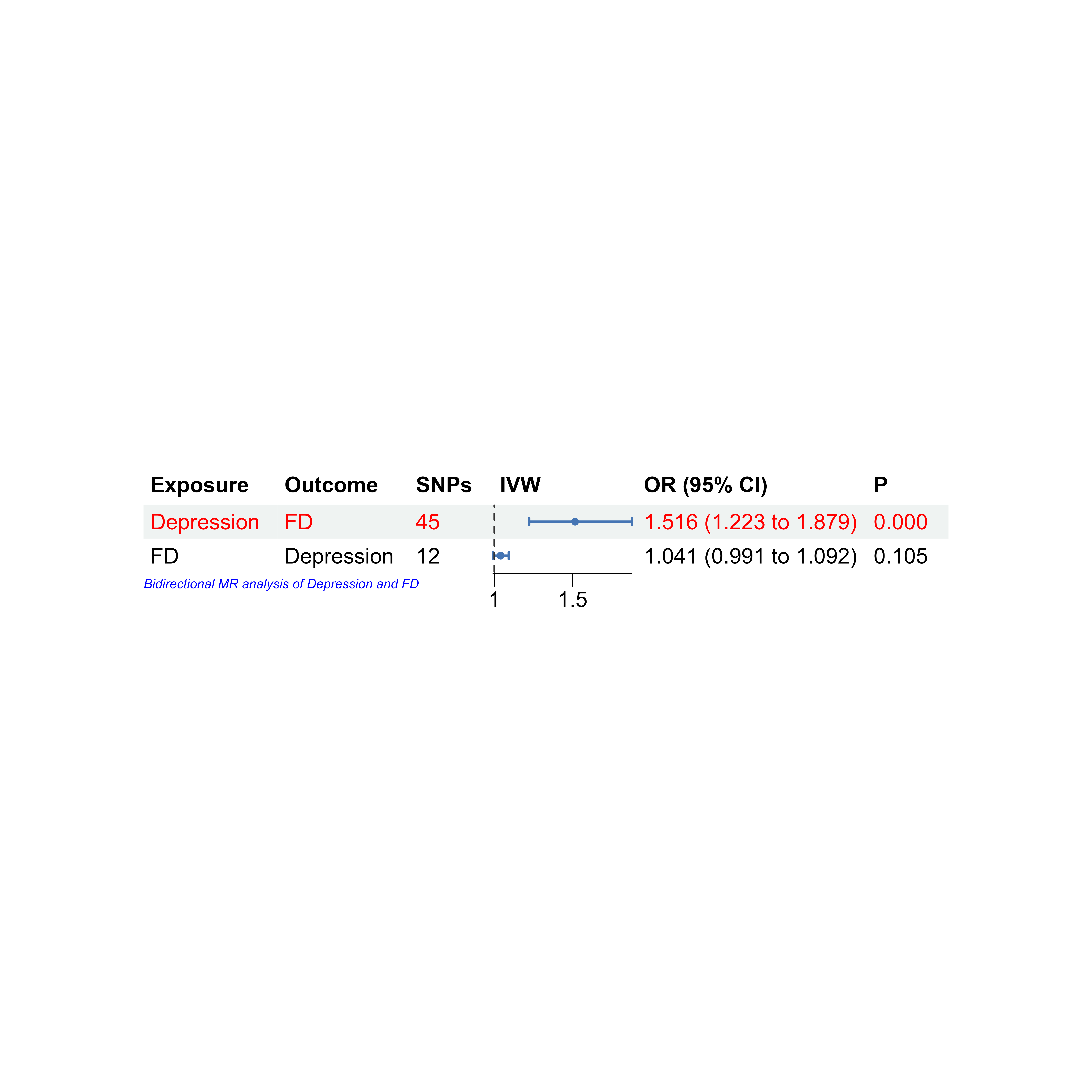

Supplement: S5 Fig — (PNG) [file pone.0302809.s005.png]
