# Supplementary material for: The associations between functional dyspepsia and potential risk factors: A comprehensive Mendelian randomization study
Source: PLoS One. 2024 May 8;19(5):e0302809. doi: 10.1371/journal.pone.0302809 (PMC11078438; doi:10.1371/journal.pone.0302809)
Supplement: S2 File — (DOCX) [file pone.0302809.s007.docx]

S1 Table: Possible risk factors for the risk of FD identified in the previous meta-analyses or reviews.

| **Author (year)** | **Study design** | **Risk factors** | **PMID** |
| --- | --- | --- | --- |
| Yan (2023) | Meta-analysis | Anxiety, depression, mental disorder, somatization, sleep disorder | 36894717 |
| Popa (2022) | Review | High-fat food | 35631198 |
| Lane (2021) | Meta-analysis | Ultraprocessed food | 33167080 |
| Talley (2021) | Meta-analysis | Smoking | 33983640 |
| Watanabe (2021) | Review | CRP | 35832706 |
| Ding (2020) | Review | NAFLD | 33177790 |
| Ford. (2020) | Review | Female gender, smoking, use of non-steroidal anti-inflammatory drugs, H.pylori infection, higher body mass index, anxiety, depression, acute gastroenteritis, high fat foods, heavy chilli intake, lower socioeconomic status. | 33049222 |
| Talley (2020) | Review | Infection, diet, H.pylori infection, smoking, anxiety, depression, somatization, higher levels of psychological distress | 31895721 |
| McCormick (2019) | Review | Constipation | 31002490 |
| Cryan. (2019) | Review | Microbiota | 31460832 |
| Bharucha (2019) | Review | Diabetes | 31081877 |
| Duncanson (2018) | Review | Wheat and dietary fats | 28913843 |
| Talley (2017) | Review | Postinfectious gastroenteritis | 28452210 |
| Ohlsson (2017) | Review | Smoking, high alcohol intake | 29195674 |
| Tan (2017) | Review | Diet | 28244670 |
| Talley (2016) | Review | Possibly food, helicobacter pylori infection, GNbeta3 | 27048251 |
| Talley (2015) | Review | Upper intestinal inflammation, G-protein beta 3 subunit 825, acute infection, anxiety, aspirin | 26444826 |
| Ford (2015) | Meta-analysis | Female gender, smoking, non-steroidal inflammatory drug use and H. pylori | 25147201 |
| Yap (2015) | Review | NSAIDs | 26369685 |
| Talley (2015) | Review | IBS | 25917563 |
| Quigley (2013) | Review | GERD | 23296247 |
| Pike (2013) | Meta-analysis | Acute gastroenteritis | 23711623 |
| Miwa (2011) | Review | Motility abnormalities, visceral hypersensitivity, psychosocial factors, excessive gastric acid secretion, Helicobacter pylori, genetics, environment, diet, lifestyle, post-infectious FD. | 21443711 |
| Ebert (2010) | Review | Thyroid disease | 20351569 |
| Mahadeva (2006) | Review | Females, underlying psychological disturbances, poor socio-economic status, smoking, increased caffeine intake, NSAIDS | 16718749 |
| Ofman (2003) | Meta-analysis | indomethacin, meclofenamate, piroxicam | 12910557 |

S2 Table: The p_pleiotropy_ and selection crieteria of each exposure

| Exposure | P_pleiotropy_ | Selection criteria |
| --- | --- | --- |
| **Hormone related factors** |  |  |
| Testosterone levels | 0.5772 | p< 5×10^–8^ |
| Estradiol levels | NA | p< 5×10^–8^ |
| **Medication use factors** |  |  |
| Medication use (antihypertensives) | 0.6996 | p< 5×10^–8^ |
| Medication use (antidepressants) | NA | p<1×10^–5^ |
| Treatment/medication code: aspirin | 0.9772 | p< 5×10^–8^ |
| Treatment/medication code: ibuprofen | 0.5909 | p< 5×10^–8^ |
| Treatment/medication code: paracetamol | 0.0639 | p< 5×10^–8^ |
| Treatment/medication code: meloxicam | 0.1915 | p<1×10^–5^ |
| Treatment/medication code: naproxen | 0.9250 | p<1×10^–5^ |
| Number of treatments/medications taken | 0.3580 | p< 5×10^–8^ |
| **Disease-related factors** |  |  |
| Irritable bowel syndrome | 0.3876 | p< 5×10^–8^ |
| Gastroesophageal reflux disease | 0.4247 | p< 5×10^–8^ |
| Diagnoses - main ICD10: K59.0 Constipation | 0.9745 | p<1×10^–5^ |
| H.polyri infection | 0.3102 | p<1×10^–5^ |
| hyperthyroidism | 0.6264 | p< 5×10^–8^ |
| hypothyroidism | 0.5020 | p< 5×10^–8^ |
| **Metabolism-related factors** |  |  |
| Type 1 diabetes | 0.5526 | p< 5×10^–8^ |
| Type 2 diabetes | 0.6748 | p< 5×10^–8^ |
| FastingInsulin | 0.3138 | p< 5×10^–8^ |
| FastingGlucose | 0.6145 | p< 5×10^–8^ |
| BMI | 0.8267 | p< 5×10^–8^ |
| Diagnoses - secondary ICD10: E66.9 Obesity, unspecified | 0.3102 | p< 5×10^–8^ |
| Hypertension | 0.0490 | p< 5×10^–8^ |
| Systolic blood pressure | 0.4859 | p< 5×10^–8^ |
| NAFLD | 0.7082 | p< 5×10^–8^ |
| **Sociological factors** |  |  |
| Educational attainment (years of education) | 0.6451 | p< 5×10^–8^ |
| Job involves heavy manual or physical work | 0.2611 | p< 5×10^–8^ |
| Current employment status: Doing unpaid or voluntary work | NA | p< 5×10^–8^ |
| Current employment status: Unable to work because of sickness or disability | 0.2176 | p< 5×10^–8^ |
| **Lifestyle factors** |  |  |
| Type of fat/oil used in cooking: Butter | 0.3042 | p<1×10^–5^ |
| DrinksPerWeek | 0.7854 | p< 5×10^–8^ |
| Type of fat/oil used in cooking: Vegetable oil | NA | p< 5×10^–8^ |
| Type of special diet followed: Low calorie | 0.2015 | p<1×10^–5^ |
| Type of special diet followed: Gluten-free | 0.7693 | p<1×10^–5^ |
| CigarettesPerDay | 0.3538 |  |
| Type of fat/oil used in cooking: Olive oil | 0.8416 | p<1×10^–5^ |
| Type of fat/oil used in cooking: Lard | 0.3223 | p<1×10^–5^ |
| Type of special diet followed: Vegan | 0.7277 | p< 5×10^–8^ |
| Types of physical activity in last 4 weeks: Light DIY (eg: pruning, watering the lawn) | 0.2150 | p< 5×10^–8^ |
| Types of physical activity in last 4 weeks: Heavy DIY (eg: weeding, lawn mowing, carpentry, digging) | 0.4895 | p< 5×10^–8^ |
| Poultry intake | 0.9670 | p< 5×10^–8^ |
| Type of special diet followed: Vegetarian | 0.1807 | p<1×10^–5^ |
| Type of special diet followed: Lactose-free | NA | p< 5×10^–8^ |
| **Psychological factors** |  |  |
| Autism Spectrum Disorder | NA | p< 5×10^–8^ |
| Bipolar disorder | 0.3308 | p< 5×10^–8^ |
| Anxiety | 0.5000 | p< 5×10^–8^ |
| Miserableness | 0.0152 | p< 5×10^–8^ |
| Mood swings | 0.8122 | p< 5×10^–8^ |
| Depression | 0.0018 | p< 5×10^–8^ |
| Attention Deficit Hyperactivity Disorder | 0.4449 | p< 5×10^–8^ |
| Sleep disorders | 0.6996 | p<1×10^–5^ |
| **Others** |  |  |
| Overall health rating | 0.2828 | p< 5×10^–8^ |
| C-reactive protein levels | 0.6766 | p< 5×10^–8^ |

S3 Table: Multivariate Mendelian randomization analyses for identifying the risk factors for the risk of FD.

| Exposure | method | OR (95% CI) | P |
| --- | --- | --- | --- |
| Education | MVMR_IVW | 0.907(0.839 to 0.980) | 0.014 |
| Depression | MVMR_IVW | 1.632(1.175 to 2.266) | 0.003 |
| GERD | MVMR_IVW | 0.854(0.624 to 1.169) | 0.325 |
